# Knee-ankle joint line angle: a significant contributor to high-degree knee joint line obliquity in medial opening wedge high tibial osteotomy

**DOI:** 10.1186/s13018-022-02976-y

**Published:** 2022-02-05

**Authors:** Tzu-Hao Tseng, Han-Ying Wang, Shi-Chien Tzeng, Kuan-Hung Hsu, Jyh-Horng Wang

**Affiliations:** Department of Orthopaedic Surgery, National Taiwan University Hospital, 7 Chungsan South Road, Taipei City, 10002 Taiwan

**Keywords:** Knee joint obliquity, High tibial osteotomy, Ankle, Cartilage, Correction

## Abstract

**Background:**

Medial opening wedge high tibial osteotomy (MOWHTO) changes the knee joint inclination in the coronal plane, which can be compensated by the ankle joint. Once there is a decompensated knee joint obliquity, it can induce excessive shear force on the articular cartilage. This study aimed to investigate the capacity of the compensation by analyzing the correlation of the knee-ankle joint line angle (KAJA) and the knee joint line obliquity (KJLO).

**Patients and Methods:**

Ninety-six patients undergoing MOWHTO were included. We measured potential predictors including preoperative or postoperative body mass index (BMI), weight-bearing line (WBL) ratio/correction amount, knee-ankle joint line angle(KAJA), mechanical lateral distal femoral angle (mLDFA), medial proximal tibia angle (MPTA), ankle joint line obliquity (AJLO), mechanical hip-knee-ankle angle (mHKA) and joint line convergence angle (JLCA). The correlations of these predictors and postoperative KJLO were determined using Pearson correlation coefficient. The contribution of significant predictors was further analyzed using multiple linear regression. Finally, the cutoff value of the most contributing factor resulting in decompensated KJLO was derived with receiver operating characteristic (ROC) curve analysis.

**Results:**

Preoperative AJLO, JLCA, MPTA, mHKA and KJLO and postoperative KAJA and MPTA correlated with postoperative KJLO. After multiple linear regression, only preoperative AJLO and JLCA and postoperative KAJA still showed significant contribution to postoperative KJLO. Postoperative KAJA made the greatest contribution. The cutoff value of postoperative KAJA was at 9.6° after ROC analysis. The incidence rate of high-grade KJLO was 69.6% when postoperative KAJA exceeded 9.6°.

**Conclusions:**

Postoperative KAJA is a significant contributor to high-grade KJLO after MOWHTO. The incidence was increased at angles greater than 9.6°. The results suggest that KAJA should be carefully assessed during preoperative planning or intraoperative evaluation. Postoperative KAJA < 9.6° can lower the rate of early high-degree KJLO.

## Introduction

Medial opening wedge high tibial osteotomy (MOWHTO) is a commonly performed procedure for medial osteoarthritis of the knee [1–3]. To relieve stress in the medial compartment, MOWHTO shifts the mechanical axis of the lower extremity laterally. However, this procedure inevitably changes the joint inclination along the coronal plane because the medial tibia is elevated [4–7]. In cases of severe varus deformity of the knee joint due to proximal tibial deformity or combined deformity of both the distal femur and proximal tibia, single-level osteotomy can result in high-degree knee joint line obliquity (KJLO) because a large amount of correction is required [4, 5]. The acceptable limit of KJLO appears to be approximately 5° [8, 9]. Excessive KJLO leads to worse radiographic and clinical outcomes [8]. The shear force in the medial compartment increases significantly [9], which can subsequently cause chondrocyte death [10–12].

Since KJLO cannot be assessed accurately intraoperatively, there have been several studies that discuss preoperative parameters associated with greater postoperative KJLO [7, 13]. Preoperative mechanical hip-knee-ankle angle (mHKA), medial proximal tibial angle (MPTA) and joint line convergence angle (JLCA) are well-established predictors of postoperative abnormal joint line obliquity. Previous studies explained that these parameters can predict abnormal obliquity since large corrections were necessitated. However, in some cases, we observed that after MOWHTO, even a relatively small correction could result in high-degree KJLO. Therefore, there must be other contributing factors that have not been identified yet.

Postoperative knee joint obliquity can be compensated by the ankle joint [6, 14–16]. The mobility of the subtalar joint, which differs per individual, determines the capacity of the ankle joint to compensate [17, 18]. Therefore, we speculated that the compensation achieved by the ankle joint was limited. Exceeding these limits may result in an unacceptable rate of high-degree KJLO, even after a small correction. This study aimed to investigate the correlation between the knee-ankle joint line angle (KAJA) and KJLO. The hypothesis of our study was that higher postoperative KAJA would result in higher KJLO, and there would be a critical value of KAJA above which KJLO could not be well compensated.

## Methods

We conducted a retrospective observational study at the corresponding author’s hospital. The study was approved by the Ethics Committee of the hospital, and a waiver of informed consent for the retrospective use of patient data (approval number: 201910040RIND) was obtained. We investigated 111 consecutive patients who underwent one-sided MOWHTO between January 2016 and April 2019. The indications for MOWHTO were as follows: symptomatic medial unicompartmental osteoarthritis with a mechanical tibiofemoral angle of at least 5° and flexion contracture of less than 10°. Fourteen patients were excluded due to a lack of preoperative or postoperative standing anteroposterior radiographs of the lower extremities in the picture archiving and communication system (PACS) of the hospital. One patient was excluded because proximal fibulectomy was performed concurrently with MOWHTO. A total of 96 patients, composed of 35 men and 61 women, were finally included, with a mean age of 60.0 ± 8.5 years (mean ± standard deviation, SD). None of patients had preoperative ankle symptom.

### Surgical technique and rehabilitation protocol

We performed an arthroscopic examination before the MOWHTO, and arthroscopic drilling was performed if there was subchondral bone exposure. After arthroscopy, we made a skin incision on the anteromedial aspect of the tibia. Generally, the detailed steps were the same as those described previously [19, 20]. Biplanar osteotomy was performed with a transverse osteotomy from the medial tibial cortex to the lateral tibial cortex just above the proximal tibiofibular joint and a ventral ascending osteotomy in the coronal plane. We used a locking plate designed for the medial proximal tibia to fix the biplanar MOWHTO. The procedure aimed to align the weight-bearing line through the Fujisawa point [21, 22]. Intraoperatively, we used “the alignment rod” method [23] to check for correction.

Partial weight bearing was allowed immediately after surgery, and full-weight bearing was started 4 weeks later. Early muscle strengthening and range of motion exercises were taught by physiotherapist since postoperative day 2. The rehabilitation program was then individualized according to the patients’ fitness and their target activity level. Generally, the goal was to return to their previous activity level between 3 and 6 months.

### Potential predictors by radiological evaluations

Before surgery, all patients underwent bilateral leg standing radiography. After surgery, bilateral leg standing radiographs were obtained when the patient could fully bear their body weight without discomfort, which was between 6 weeks and 3 months after MOWHTO.

The potential predictors are radiographic parameters centered around the knee joint. These included the MPTA, KAJA, mHKA, mechanical lateral distal femoral angle (mLDFA), weight-bearing line (WBL) ratio, KJLO and ankle joint line obliquity (AJLO). In addition to the preoperative values, we also included postoperative values if the parameter could be determined by a single bone, which would not be affected by weight-bearing status and could be theoretically simulated during preoperative planning (e.g., KAJA and MPTA). In contrast, the parameters involving different bones were measured preoperatively only (e.g., mHKA, WBL ratio, AKLO and KJLO). The postoperative values of these parameters were affected by different soft tissue condition before and after MOWHTO, which was unpredictable before the procedure.

The potential predictors were measured on bilateral leg standing radiographs, and the measurement methods are described as follows:

1. MPTA: This was the medial angle between the mechanical axis of the tibia and articular surface of the proximal tibia (Fig. 1a).

**Fig. 1:**
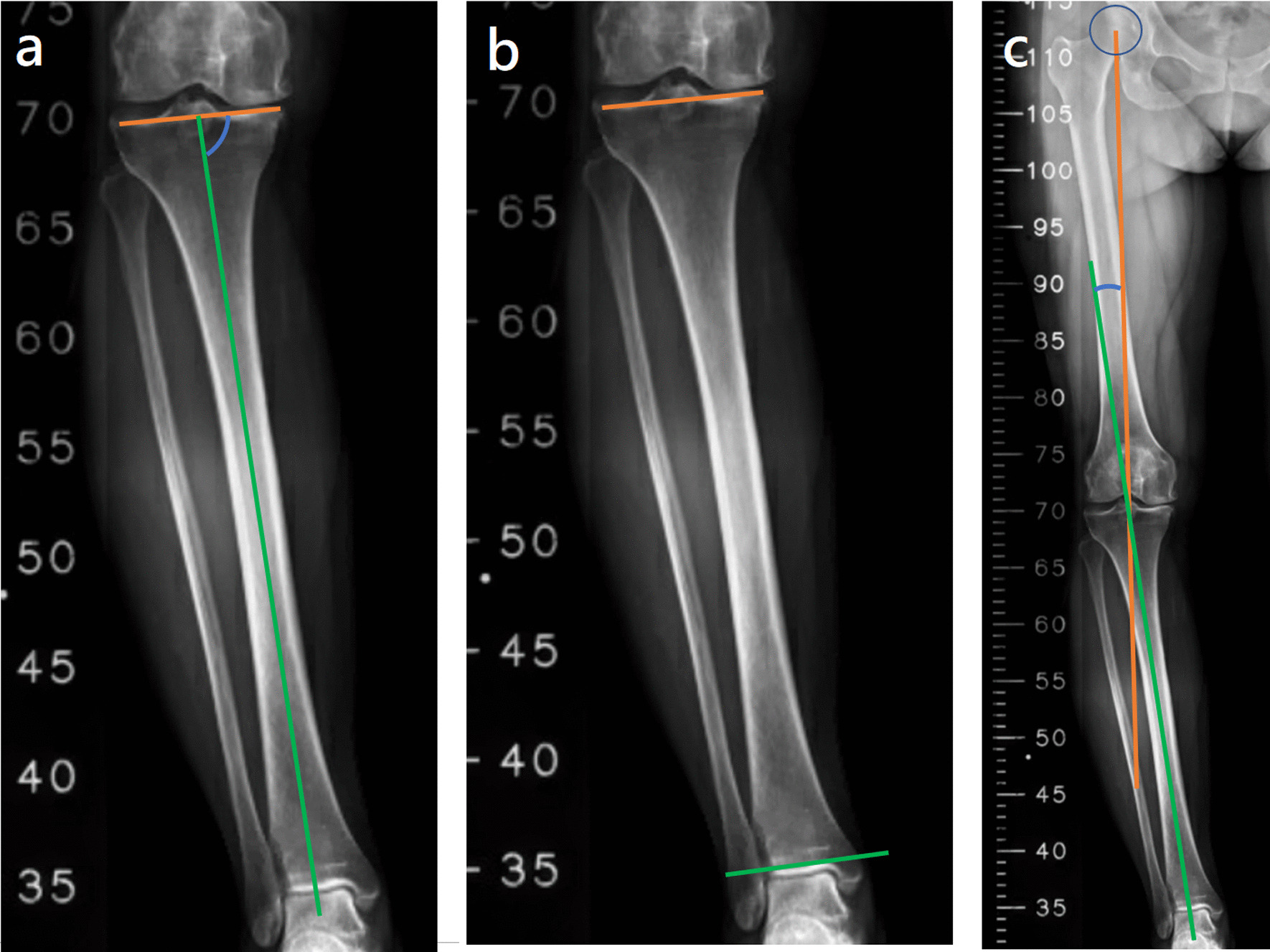
**a** MPTA: the medial angle between the mechanical axis of the tibia (indicated by the green line) and the articular surface of the proximal tibia (indicated by the orange line), **b** KAJA: the angle between the lines tangent to the articular surfaces of the proximal tibia (indicated by the orange line) and distal tibia(indicated by the green line), **c** mHKA: the angle between the mechanical axes of the femur (indicated by the orange line) and tibia (indicated by the green line)
2. KAJA: This was the angle between the lines tangent to the articular surfaces of the proximal tibia and distal tibia. A positive value represented a valgus relationship between these two surfaces, and a negative value represented a varus relationship (Fig. 1b).
3. mHKA: This was the angle between the mechanical axes of the femur and tibia (Fig. 1c).
4. mLDFA: This was the lateral angle formed between the femoral mechanical axis and joint line of the distal femur (Fig. 2a).

**Fig. 2:**
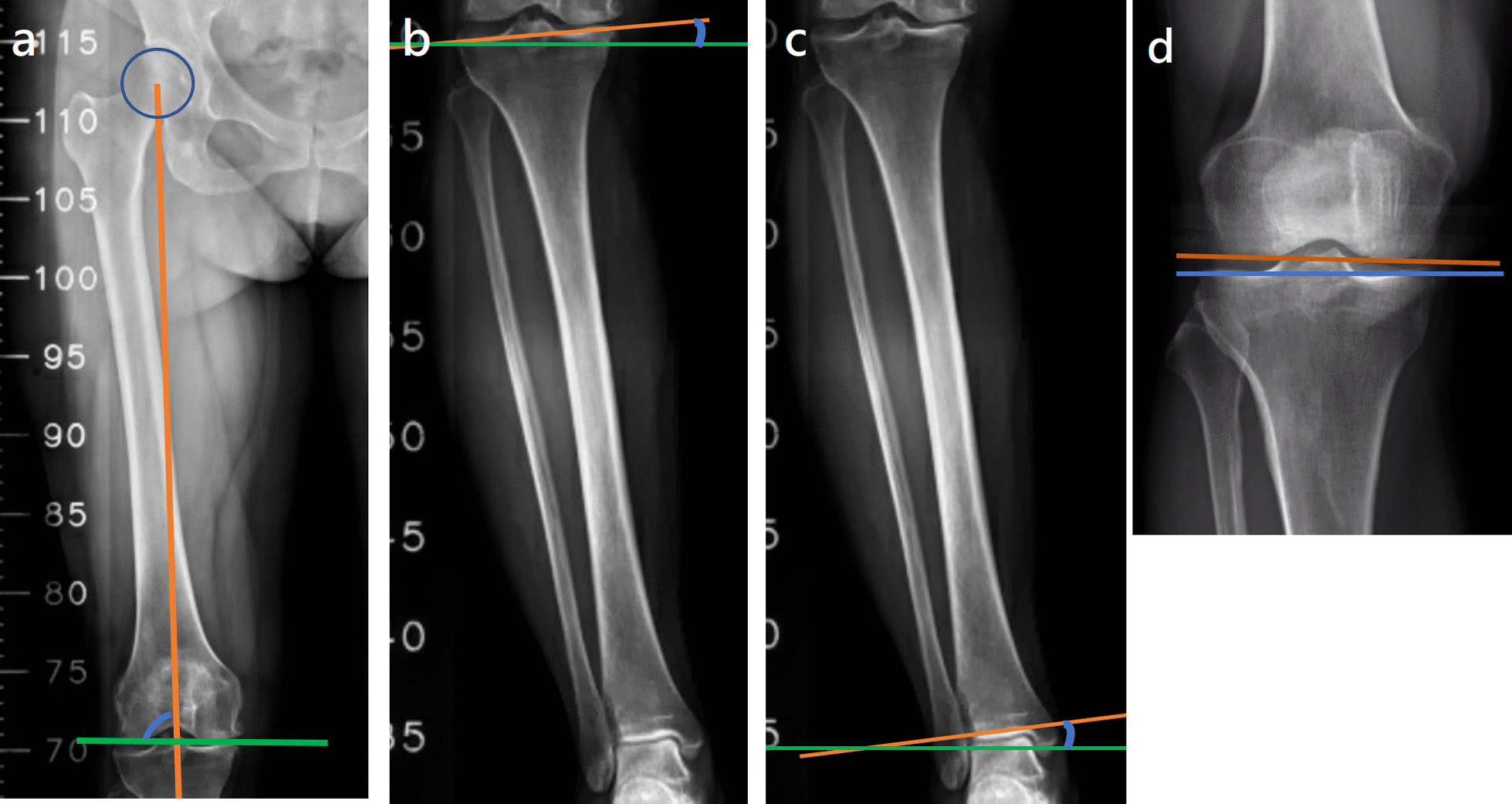
**a** mLDFA: the lateral angle formed between the femoral mechanical axis (indicated by the orange line) and the joint line of the distal femur (indicated by the green line), **b** KJLO: the angle between a line tangent to the articular surface of the proximal tibia (indicated by the orange line) and a line parallel to the ground (indicated by the green line), **c** AJLO: the angle between a line tangent to the articular surface of the distal tibia (indicated by the orange line) and a line parallel to the ground (indicated by the green line). **d** JLCA: the angle between a line tangent to the articular surface of the distal femur (indicated by the orange line) and a line tangent to the articular surface of the proximal tibia (indicated by the blue line)
5. WBL ratio: This was determined by the intersection of the articular surface of the proximal tibia and a line from the center of the femoral head to the center of the talus. The ratio was obtained by dividing the distance measured from the edge of the medial tibial plateau by the total width of the articular surface. The discrepancy between preoperative and postoperative WBL ratios was defined as the correction amount.
6. KJLO: This was the angle between a line tangent to the articular surface of the proximal tibia and a line parallel to the ground. A positive value indicated that the medial articular surface is higher than the lateral surface (Fig. 2b). A KJLO ≥ 5° was defined as a high-degree KJLO. We divided the KAJA by 5° based on a previous biomechanical study [9] and a clinical study [8].
7. AJLO: This was the angle between a line tangent to the articular surface of the distal tibia and a line parallel to the ground. A positive value indicated that the medial articular surface was higher than the lateral surface (Fig. 2c).

All the potential predictors were measured by three authors in a blinded manner. One of them is an orthopedic surgeon, and the other two are nurse practitioners who participates in orthopedic surgeries as daily work. All of them measured the angles again in a blinded manner more than 4 weeks later.

### Statistical analysis

All statistical analyses were performed using SPSS version 20 (IBM Corp., Armonk, NY, USA) on a Microsoft Windows-based computer. The correlation between continuous data was determined using Pearson’s correlation coefficient. Setting the postoperative KJLO as the dependent variable and all the above potential radiographic parameters, sex and body mass index (BMI) as independent variables, multiple linear regression analysis was performed. The cutoff value for the factor contributing the most to high-degree KJLO was derived using the receiver operating characteristic (ROC) curve. The interobserver and intraobserver reliabilities of the measured angles were assessed using the intraclass correlation coefficient (ICC). Statistical significance was set at a *P*-value of < 0.05. Post hoc power analysis was performed using the G*Power software (version 3.1.9.4) for Pearson’s correlation coefficient and multiple linear regression analysis, and Medcalc software (version 20.023) for ROC curve.

## Results

There were no peri-implant fractures or infections that could influence KJLO. The preoperative and postoperative WBL ratios were 15.3% ± 15.4% and 63.3% ± 15.1%, respectively. In all cases, the KAJA increased after MOWHTO. The mean value of each potential predictor and its correlation with the postoperative KJLO are shown in Table 1.

**Table 1:** The mean value of radiographic parameters and their correlations with postoperative KJLO

	Mean ± SD	Pearson correlation coefficient	*P*-value
BMI	27.0 ± 3.8	− 0.093	0.367
Pre-KJLO	− 0.7 ± 3.3	0.484	< 0.001
Pre-KAJA	3.2 ± 4.4	0.112	0.279
Post-KAJA	− 5.7 ± 4.6	0.504	< 0.001
mLDFA	90.2 ± 2.8	0.094	0.366
Pre-MPTA	84.7 ± 2.9	0.252	0.013
Post-MPTA	93.9° ± 3.0°	0.466	< 0.001
Pre-AJLO	2.5 ± 5.0	0.221	0.031
Pre-mHKA	7.4 ± 3.7	0.322	0.001
Pre-JLCA	3.0° ± 1.6°	0.544	< 0.001
Correction amount	48.2 ± 16.9	0.012	0.914

Pre-, preoperative; post-, postoperative; BMI, body mass index; KJLO, knee joint line obliquity angle; KAJA, knee-ankle joint angle; mLDFA, mechanical lateral distal femoral angle; MPTA, medial proximal tibial angle; AJLO, ankle joint line obliquity angle; mHKA, mechanical hip-knee-ankle angle; and JLCA, joint line convergence angle

The postoperative KAJA (Pearson’s correlation coefficient: 0.504, *P* < 0.001) and preoperative KJLO (Pearson’s correlation coefficient: 0.484, *P* < 0.001) were moderately correlated with postoperative KJLO. Preoperative MPTA (Pearson’s correlation coefficient: 0.252, *P* = 0.013), mHKA (Pearson’s correlation coefficient: 0.322, *P* = 0.001), AJLO (Pearson’s correlation coefficient: 0.221, *P* = 0.031) were weakly correlated with postoperative KJLO. The Pearson’s correlation coefficient between each potential predictor and postoperative KJLO is shown in Table 1. Post hoc power for Pearson’s correlation coefficient was 0.92 (medium effect size at 0.3, alpha error at 0.05 and total sample size at 96).

After multiple linear regression, the *F*-value was 52.548 (*P* < 0.001) and the R^2^ value was 0.784. Only preoperative AJLO, JLCA and postoperative KAJA still showed a significant contribution to postoperative KJLO. Postoperative KAJA contributed the most. Other potential predictors were excluded from the stepwise regression analysis. The results of the multiple linear regression are shown in Table 2. Post hoc power for multiple linear regression was 1.00 (R^2^ at 0.784, alpha error at 0.05, total sample size at 96 and number of predictors at 12).

**Table 2:** Model after multiple linear regression

	*β*-coefficient	*t*-value	*P*-value	VIF
Post-KAJA	0.466	4.894	< 0.001	1.494
Pre-AJLO	0.290	3.271	0.002	1.297
Pre-JLCA	0.278	2.971	0.004	1.445
*Excluded variables*				
Sex	0.050	0.596	0.553	1.020
BMI	0.051	0.610	0.543	1.005
Pre-KJLO	0.136	1.260	0.211	1.927
Pre-KAJA	0.005	0.054	0.957	1.040
mLDFA	0.104	1.261	0.211	1.000
Pre-MPTA	0.076	0.906	0.367	1.159
Post-MPTA	0.149	1.559	0.123	1.530
Pre-mHKA	0.145	1.620	0.109	1.343
Correction amount	0.035	0.330	0.742	1.615

Pre-, preoperative; post-, postoperative; BMI, body mass index; KJLO, knee joint line obliquity angle; KAJA, knee-ankle joint angle; mLDFA, mechanical lateral distal femoral angle; MPTA, medial proximal tibial angle; AJLO, ankle joint line obliquity angle; mHKA, mechanical hip-knee-ankle angle; and JLCA, joint line convergence angle

The factor contributing the most to high-degree KJLO, postoperative KAJA, was further analyzed using ROC curve analysis. The area under the ROC curve was 0.834 ± 0.056 (*P* < 0.001), with the highest Youden’s index of 1.60 occurring at 9.6°. The incidence rate of high-grade KJLO was 69.6% (16/23 knees) when the postoperative KAJA (sensitivity, 0.70; specificity, 0.90) was ≥ 9.6°, whereas the rate was only 9.6% when postoperative KAJA was < 9.6° (7/73 knees). ICC of intraobserver and interobserver agreement for radiologic evaluations was all acceptable at > 0.87 (range 0.87–0.98). Post hoc power for ROC curve was > 0.99 (alpha error at 0.05, area under curve at 0.834, null hypothesis value at 0.5, ratio of negative/positive sample sizes at 73/23 = 3.174).

## Discussion

The most important finding of this study was that postoperative KAJA is significantly correlated with KJLO. The contribution of KAJA was stronger than that of the previously reported factors. The rate of ≥ 5° KJLO was 69.6% when postoperative KAJA exceeded 9.6°. Therefore, the postoperative KAJA should be below 9.6° for postoperative KJLO to remain below 5 degrees. Double-level osteotomy may be a solution if optimal alignment cannot be achieved [24].

The upper limit of an acceptable KJLO is defined as approximately 5° in previous studies. KJLO > 6° and KJLO > 4° lead to worse radiographic and clinical outcomes, respectively [8]. Nakayama et al. also showed that the shear force in the medial compartment was almost twice as high as that in the normal knee when the obliquity angle is 5° [9]. The force value becomes even higher as the obliquity angle increases. Although some studies have shown that there is no difference in short-term outcomes with high-degree obliquity [6, 25, 26], it is reasonable that there would be a long-term adverse effect on the articular cartilage [8, 11]. Lee et al. found that the change in KJLO was significantly less than that of the anatomical geometry of the proximal tibia. This phenomenon can be explained by the compensation of the ankle joint [6, 16]. However, the capacity for compensation differs among individuals [7, 14, 27], and currently, there is no information about the maximum of the capacity for most patients undergoing MOWHTO. Understanding when the knee obliquity will be “decompensated” is essential to ensure a satisfactory outcome. Since KJLO cannot be obtained until postoperatively, the KAJA appears to be a reasonable parameter when analyzing this issue. In this study, the area under the ROC curve was 0.834, indicating good discrimination of the KAJA. The highest Youden’s index occurred at 9.6°. The rate of high-degree KJLO became unacceptably high when KAJA was larger than 9.6°. Therefore, these results suggest that the change of the anatomical geometry of the proximal tibia caused by MOWHTO could not be sufficiently compensated by the mobility of the subtalar joint once the KAJA exceeded 9.6°. When performing the MOWHTO, a 9.6° KAJA should be considered as a critical value.

In accordance with previous studies, higher preoperative mHKA and MPTA were correlated with higher postoperative KJLO [7, 13]. It is reasonable that a higher mHKA might require a larger correction, which is supposed to result in greater obliquity. However, the correction amount evaluated as the change in the WBL ratio was not a significant factor. The postoperative mean WBL ratio was 63.3% in this study, which is remarkably close to the classic aim at 62.5% [21, 22]. Therefore, when correcting a lower limb from a higher mHKA to the classic surgical goal, adherence of the WBL to the Fujisawa point would tend to result greater postoperative obliquity. We speculated that the change in the WBL ratio could be affected by multiple factors, such as anatomical variances of the femur and tibia. Thus, it does not necessarily represent a large angle correction. In contrast, a higher preoperative mHKA may be a more representative factor. Furthermore, a higher preoperative MPTA implied that the proximal tibia contributed less to lower extremity varus deformity. Therefore, MOWHTO on knees with higher MPTA may result in a less physiologically oriented joint line.

**Fig. 3:**
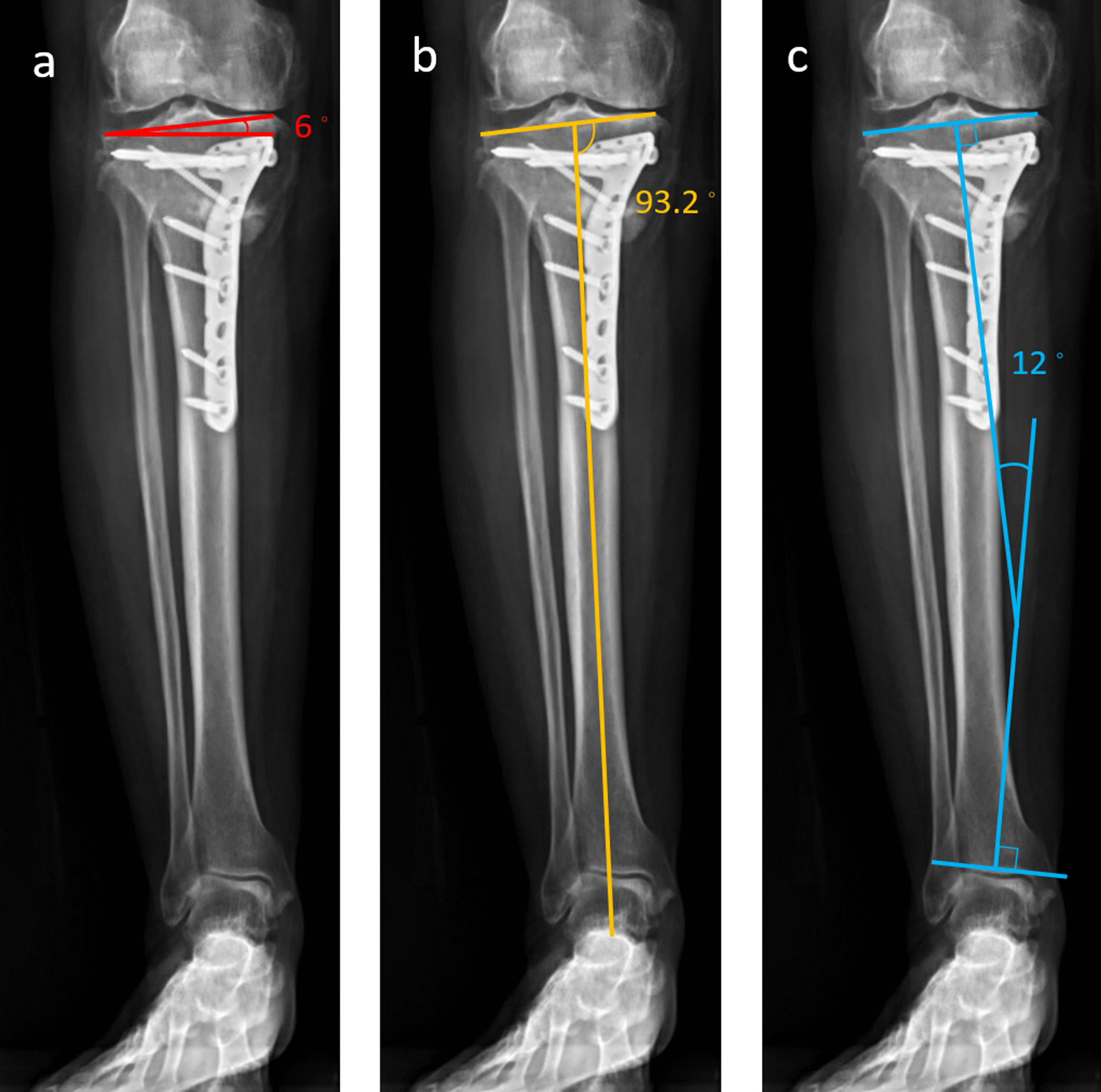
An example of high-degree KJLO with high-degree KAJA and acceptable MPTA. **a** KJLO: 6°, **b** MPTA: 93.2° (lower than the mean value in this study), **c** KAJA: 12° (higher than 9.6°)

In all cases, the KAJA increased after MOWHTO. Although postoperative KAJA contributed the most to postoperative KJLO, preoperative KAJA did not correlate with it. Similarly, after multiple linear regression, preoperative KJLO did not contribute to high-degree KJLO. In other words, a high postoperative KAJA is the true contributing factor of high-degree KJLO after eliminating the confounding effects. An example of high-degree KJLO with high KAJA and acceptable MPTA is shown in Fig. 3. These results suggest that the KAJA should be assessed carefully during preoperative planning or intraoperatively to avoid exceeding the critical 9.6° KAJA. Because of the retrospective nature of this study, it could not be determined if the postoperative KAJA could simply be calculated by adding the preoperative KAJA to the intraoperative correction angle (e.g., the osteotomy angle). We recommend assessing the intraoperative KAJA by measuring the angles between the tibial articular surfaces and a reference rod at the knee and ankle joints. KJLA can be calculated by subtracting these two angles. These measurements are not burdensome to perform because they can be performed along with the assessment of the mechanical axis by the “cable method” [28] or “alignment rod” method [23].

The limitations of this study are as follows. First, avoiding ≥ 9.6° KAJA did not prevent all high-degree KJLO. However, the incidence rate of high-degree KJLO was only 9.6% when KAJA was below 9.6°. Second, this study only focused on KJLO. Whether compensation caused further adverse effects on the ankle joint was not clear. A prospective long-term study is needed to clarify this issue. Third, since the capacity for the ankle joint compensation differs across individuals, the health status of the subtalar joint may play an important role in KJLO after MOWHTO. However, none of the patients had preoperative ankle symptom. Fourth, this study only confirmed the relationship between potential predictors and early postoperative KJLO. The soft tissue conditions around the knee may change with time. Therefore, the correlation analysis at different time points may produce different results. However, the present findings could help prevent obliquity in the early postoperative period. Further studies are necessary to identify the clinical benefits of preventing early KJLO. Furthermore, because the follow-up time was still short for most patients and the purpose of this retrospective study was to identify the predictors of postoperative KJLO only, we could not provide radiographic and clinical outcomes. Previous studies have reported the adverse effects of high KJLO on radiographic and clinical outcomes after MOWHTO [8, 9].

## Conclusion

Postoperative KAJA is a significant contributor to high-grade KJLO after MOWHTO. The incidence was increased at angles greater than 9.6°. The results suggest that KAJA should be carefully assessed during preoperative planning or intraoperative evaluation. Postoperative KAJA < 9.6° can lower the rate of early high-degree KJLO.

## Abbreviations

AJLO: Ankle joint line obliquity
BMI: Body mass index
ICC: Intraclass correlation coefficient
JCLA: Joint line convergence angle
KAJA: Knee-ankle joint line angle
KJLO: Knee joint line obliquity
mHKA: Mechanical hip-knee-ankle angle
mLDFA: Mechanical lateral distal femoral angle
MOWHTO: Medial opening wedge high tibial osteotomy
MPTA: Medial proximal tibial angle
ROC: Receiver operating characteristic
WBL: Weight-bearing line
